# Evaluation of annexin A5 as a biomarker for Alzheimer's disease and dementia with lewy bodies

**DOI:** 10.3389/fnagi.2013.00015

**Published:** 2013-04-05

**Authors:** Hitoshi Sohma, Shin-ichi Imai, Norio Takei, Hirohito Honda, Kyoichi Matsumoto, Kumiko Utsumi, Kayo Matsuki, Eri Hashimoto, Toshikazu Saito, Yasuo Kokai

**Affiliations:** ^1^Department of Educational Development, Center for Medical Education, Sapporo Medical UniversitySapporo, Japan; ^2^Department of Biomedical Engineering, School of Medicine, Sapporo Medical UniversitySapporo, Japan; ^3^Mikuri Immunology Laboratory, Inc.Osaka, Japan; ^4^Sanyo Chemical Industries, Ltd.Kyoto, Japan; ^5^Department of Neuropsychiatry, Sunagawa City Medical CenterSunagawa, Japan; ^6^Department of Neuropsychiatry, School of Medicine, Sapporo Medical UniversitySapporo, Japan

**Keywords:** plasma biomarker, Alzheimer's disease, dementia with lewy bodies, annexin A5, Ca^2+^-stress, ROC curve, ApoE

## Abstract

**Background:** Alzheimer's disease (AD) differs from other forms of dementia in its relation to amyloid beta peptide (Aβ_42_). Using a cell culture model we previously identified annexin A5, a Ca^2+^, and phospholipid binding protein, as an AD biomarker. Plasma level of annexin A5 was significantly higher in AD patients compared to that in a control group. On the other hand, AD has been identified to share a number of clinical and pathological features with Dementia with Lewy bodies (DLB). The present study was done to examine whether or not plasma annexin A5 is a specific marker for AD, when being compared with the levels of DLB patients. As Apolipoprotein E (ApoE) gene subtype ε4 (ApoE-ε4) has been noticed as the probable genetic factor for AD, we also examined and compared ApoE genotype in both AD and DLB.

**Methods:** Blood samples were obtained from 150 patients with AD (aged 77.6 ± 6.5 years), 50 patients of DLB (79.4 ± 5.0) and 279 community-dwelling healthy elderly individuals of comparable age and sex (75.6 ± 8.1). All AD patients met NINCDS-ADRDA criteria and all DLB patients were diagnosed as probable DLB according to the latest consensus diagnostic criteria. Quantification was done using the Chemiluminescent Enzyme Immunoassay (CLEIA) Technique (SphereLight assay) using the monoclonal antibodies against annexin A5. DNA genotyping of ApoE was performed by distinguishing unique combinations of Hha1 fragments of PCR-amplified genomic DNA products.

**Results:** The plasma level of annexin A5 was significantly higher in AD patients than in the healthy individuals (control) (*P* < 0.0001). The plasma annexin A5 level was also significantly higher in DLB patients than in the control group (*P* < 0.0001). From the ROC curves with plasma annexin A5 concentrations, the mean areas under the curve were 0.863 and 0.838 for the AD/control and DLB/control, respectively. The rate of ApoE4 carrier status and the frequency of the ε4 allele were significantly higher in AD or DLB than in control and there was no significant difference between AD and DLB.

**Conclusions:** These results suggest that both annexin A5 and ApoE4 are common markers for AD and DLB.

## Introduction

The augmented number of dementia patients is remarkable in the aging of society in advanced countries. Alzheimer's disease (AD) accounts for more than half of all dementia, and Dementia with Lewy bodies (DLB) are the second most common, accounting for approximately 15% of cases at autopsy (McKeith et al., 2004), both of which are common forms of neurodegenerative dementia. DLB shares clinical and pathological features with other dementia subtypes such as AD, vascular dementia and Parkinson's disease (PD), which makes it difficult to distinguish in clinical practice. Also, the lack of valid and reliable methods for assessing the core clinical symptoms of both AD and DLB makes its identification even more difficult. The diagnosis of AD is reliant on the use of National Institute of Neurological and Communicative Disorders and Stroke-AD and related Disorders Association (NINCDS-ADRDA) criteria. The NINCDS-ADRDA criteria have high sensitivity (0.93), but low specificity (0.23) in the diagnosis of AD among a group of patients with cortical dementias [AD and frontotemporal dementia (FTD)] (Varma et al., 1999). On the other hand, consensus criteria for DLB were developed in 1996 to assist with the ante-mortem diagnosis (McKeith et al., 2005). Although the criteria have high specificity (79–100%), the sensitivity is low (20–60%), so that the diagnosis can be missed in many cases during life (Litvan et al., 2003). The revised clinical consensus criteria were published in 2005, which gives greater diagnostic weight to clinical features suggestive of DLB (McKeith et al., 2005). In light of the limited sensitivity of current methods of clinical diagnosis, it is important to establish additional markers that can improve diagnostic accuracy in combination with clinical assessment.

Amyloid β peptide (Aβ), which is a proteolytic product of amyloid precursor protein (APP), accumulates in the brains of AD patients. Its toxicity is thought to cause neural cell death (Mattson, 2004). Amyloid-dependent neurotoxicity is known to perturb Ca^2+^ homeostasis in neuronal cells (LaFerla, 2002). Possibly, Aβ impairs membrane Ca^2+^ pumps and enhances Ca^2+^ influx through voltage-dependent channels and ionotropic glutamate receptors. Focusing on this mechanism, we identified the Ca^2+^-related protein as a potential biomarker for AD using primary neurons as a cell culture model (Yamaguchi et al., 2010). It was shown that the level of annexin A5 was augmented in both the brain and blood plasma in an AD-model mouse (Tg2576 transgenic mouse), overexpressing mutant human APP (Yamaguchi et al., 2010). In addition, the plasma level of annexin A5 was significantly increased in AD patients compared to that in a control group (*p*-value of less than 0.0001 in the logistic regression analysis), suggesting that annexin A5 is a favorable marker for AD (Yamaguchi et al., 2010). As annexin A5 binds both Ca^2+^ and lipids, it might have a role to protect against Ca^2+^-induced damage. A defensive role against apoptosis by annexin A5 is also reported, in that annexin A5 plays a role in interacting with and reducing the toxicity of the amyloidogenic proteins, islet amyloid polypeptides and α-synuclein inclusion (Bedrood et al., 2009).

Apolipoprotein E (ApoE), which is a major component of lipoproteins, is comprised of 299 amino acid residues and plays a role in the metabolism and redistribution of cholesterol. ApoE mediates the uptake of lipoprotein particles in the brain via the low-density lipoprotein (LDL), receptor related protein (LRP), and the very low-density family lipoprotein receptor (VLDL) (Mahley, 1988; Paolo and Kim, 2011). The three major isoforms of ApoE, referred to as ApoE2, E3, and E4, are products of three alleles (ε2, ε3, ε4) at a single gene locus (Mahley, 1988). Three homozygous phenotypes (Apo-E2/2, E3/3, and E4/4) and three heterozygous phenotypes (Apo-E2/3, E3/4, and E2/4) arise from the expression of any two of the three alleles. The ε4 allele of the ApoE gene was identified as the strongest genetic risk factor for AD (Bertram and Tanzi, 2008). Neuropathological studies demonstrated that the frequency of the ApoE gene subtype ε4 (ApoE ε4) allele in DLB is similar to AD and that ApoE4 has also been implicated in the development of DLB (Singletona et al., 2002). We reported that ApoE4 genotypes were similar in AD and DLB, giving further evidence that the ε4 allele is a risk factor for both disorders in Japanese subjects (Kobayashi et al., 2011).

The present study was done to examine whether or not plasma annexin A5 is a specific marker for AD, in comparison with the levels of DLB patients. For that purpose, we analyzed plasma level of DLB patients and compared with those of AD patients and age-matched community dwelling healthy persons as a control. We further discuss taking ApoE4 frequencies into consideration.

## Materials and methods

### Human blood plasma

The Sapporo Medical University Ethics Committee approved human plasma studies on dementia biomarker study in 2007. Informed written consent was obtained from all subjects. All healthy volunteers and patients provided written permission. For patients with impaired cognition we obtained written permission from their family in accordance with the Declaration of Helsinki. Blood samples were obtained from 150 patients with AD (aged 77.6 ± 6.5 years), 50 patients of DLB (aged 79.4 ± 5.0 years), and 279 community-dwelling elderly individuals (healthy volunteers) of comparable age and sex (75.6 ± 8.1 years). All AD patients met NINCDS-ADRDA criteria (McKhann et al., 1984) and DLB patients were diagnosed as probable DLB according to the latest consensus diagnostic criteria (McKeith et al., 2005). The patient's clinical symptoms were evaluated using the revised Hasegawa Dementia scale (HDS-R) (Hasegawa, 1983), Mini-Mental State Examination (MMSE), and clinical dementia rating (CDR). The diagnosis of AD was also confirmed in all patients either by brain magnetic resonance imaging or single photon emission computed tomography. Blood was drawn with Venoject II vacuum tubes containing EDTA-Na (final 4.5 mM) (Terumo, Tokyo, Japan) and the plasma fraction was isolated by centrifugation at 2500 g for 15 min. This was repeated once to avoid possible cell debris in blood. Blood was centrifuged within 6 h after sampling. Plasma fractions were stored at −80°C until use.

### Quantification of plasma level of annexin A5 using sandwich cleia (Spherelight assay)

Plasma annexin A5 was quantified using the Chemiluminescent Enzyme Immunoassay (CLEIA) Technique (SphereLight assay) as described (Yamaguchi et al., 2010). Briefly, annexin A5 present in the specimen was trapped by a monoclonal antibody (mAb) against annexin A5 (clone No. 23), conjugated to a glass bead and a horseradish peroxidase (HRP)-labeled mAb against annexin A5 (clone No. 49). Unbound materials were removed by washing. The chemiluminescent reagent consists of a luminol solution that includes a phenol-derivative as an enhancer, to which a hydrogen peroxide solution was added. The HRP in the bound conjugate catalyzes the oxidation of the luminol derivative, producing light. The light signals were read by the Olympus SphereLight180 fully automated system (Olympus Optical Co., Ltd., Tokyo, Japan). The amount of HRP conjugate bound was directly proportional to the annexin A5 concentration. The required time and volume of the specimens were 20 min and 40 μl, respectively, for the SphereLight assay. The detection limit proved to be 0.16 ng/ml for annexin A5 and this system was useful to quantify plasma annexin A5 within the range of 0.16–20.0 ng/ml. Reproducible data were obtained by intra-assay and inter-assay (data not shown). Because annexin A5 is present in blood cells (Masuda et al., 2004), if a prolonged period of time has passed (longer than 12 h) after collecting blood until centrifuging, the plasma annexin A5 level increases (data not shown) due to physical damage such as temperature change, osmotic pressure change and so on. To avoid inducible leakage of annexin A5 from blood cells, all the plasma was separated by centrifugation within 6 h of sampling. The detection limit proved to be 0.16 ng/ml of annexin A5 as previously described (Yamaguchi et al., 2010). We also performed a plasma dilution test and reproducibility studies of intra-assay and inter-assay, which confirmed the assay method is reliable (Yamaguchi et al., 2010).

### Apolipoprotein E (ApoE) genotyping

DNA genotyping of ApoE was performed according to the protocol described by Hixson and Vernier (1990). Briefly, using a QIAamp DNA Blood Mini Kit (QIAGEN, Tokyo, Japan), genomic DNA was extracted from the buffy coat after centrifugation of the blood sample according to the manufacturer's instructions. The leukocyte DNA was amplified by PCR using the oligonucleotide primers, Primer 1 (59-TAAGCTTGGCACGGCTGTCCAAGGA-39), and Primer 2 (59-ACAGAATTCGCCCCGGCCTGGTACAC-39) set on common sequence parts of ApoE isoforms. The PCR products were digested with HhaI (New England Biolabs, Japan, Inc., Tokyo, Japan) and the resulting digestion fragments were separated by electrophoresis on polyacrylamide gels (SuperSepTMDNA 15% gel (Wako, Tokyo, Japan)). Each genotype of ApoE was distinguished by unique combinations of Hha1 fragment sizes in all homozygotic and heterozygotic combinations (Hixson and Vernier, 1990). After determining the ApoE genotypes, we investigated the ApoE4 carrier status and the frequency of the ε4 allele in the 279 controls, 150 AD, and 50 DLB cases.

### Statistical analysis

The mean response of each experimental group was compared with its simultaneous control by the unpaired Student's *t*-test. Analysis of variance was used to compare the mean responses of the experimental and control groups. A significant difference was set at *p* < 0.05. Logistic regression modeling was employed to construct receiver operator curves (ROC) by using JMP 9.0.0 (SAS Institute Inc., Cary, NC) to examine the plasma annexin A5 levels in diagnoses of AD and DLB. ROC curve comparisons were based on the area under the curve (AUC), SE, and the associated 95% confidence interval (CI). We subsequently calculated sensitivity of the various models using the predicted probability of each subject by logistic regression modeling with specificity of at least eighty percent. Fisher's exact tests were used to assess the frequencies of the ε4 allele between groups using JMP 9.0.0 (SAS Institute, Cary, NC, USA).

## Results

**Figure 1:**
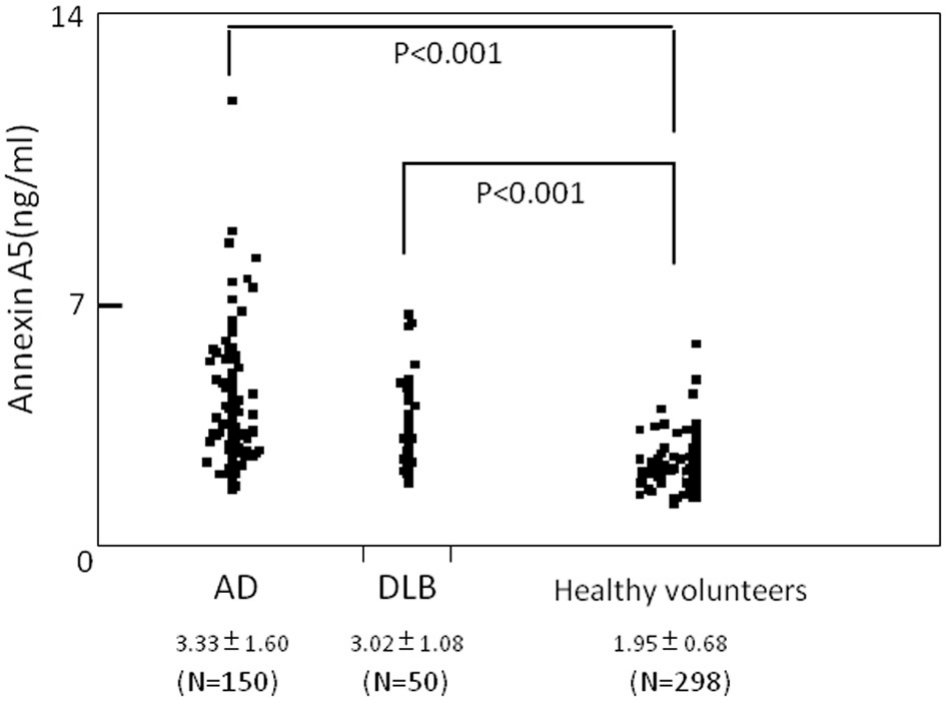
**Comparison of plasma levels of annexin A5 in AD, DLB patients, and healthy volunteers (control).** For quantitative analysis, we established a chemiluminescent enzyme immunoassay system with monoclonal antibodies against human annexin A5 and measured human plasma annexin A5. Dot blot is shown. Each point represents the plasma annexin A5 concentration of individual. AD, Alzheimer's disease; DLB, dementia with Lewy bodies.

**Figure 2:**
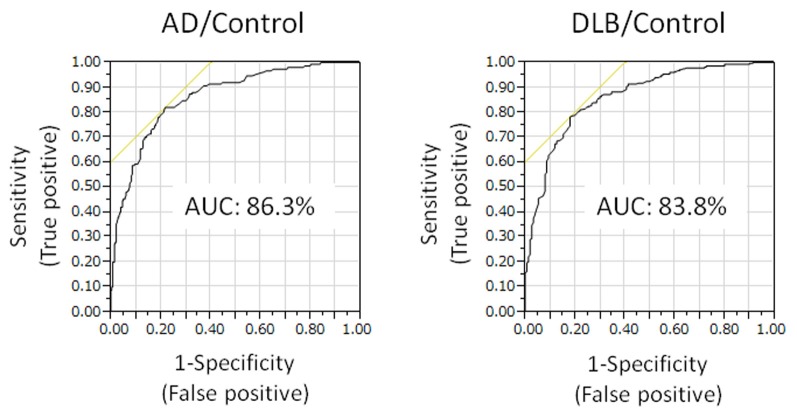
**Receiver operating characteristic (ROC) curves showing prediction of either AD or DLB by plasma annexin A5.** The probability of either AD or DLB can be predicted by a logistic regression model with the plasma level of annexin A5. The areas under the curve are 86.3 and 83.8% for AD and DLB, respectively. AD, Alzheimer's disease; DLB, dementia with Lewy bodies.

Plasma level of annexin A5 was analyzed using CLEIA Technique (SphereLight assay) as described in Materials and Methods. In this study, we measured 150 samples of AD (age 77.6 ± 6.5), 50 samples of DLB (age 79.4 ± 5.0), and 279 age-matched community dwelling healthy persons (age 75.6 ± 8.1) as a control. When average concentrations of plasma annexin A5 are compared among AD, DLB, and control groups, the values of AD (3.33 ± 1.60) and DLB (3.02 ± 1.08) were significantly higher than healthy control subjects (1.95 ± 0.68) (Figure 1). The probability of both AD and DLB can be predicted by a logistic regression model with the plasma level of annexin A5. The ROC analyses revealed good separation of patients with either AD or DLB from healthy control subjects (Figure 2). The areas under the curve were 86.3% (*P* < 0.0001) and 83.8% (*P* < 0.0001) for AD and DLB, respectively. That is statistically significant, suggesting that annexin A5 is also a potential biomarker for both AD and DLB. On the other hand, no significant difference was observed between AD and DLB (*p* = 0.36).

**Figure 3:**
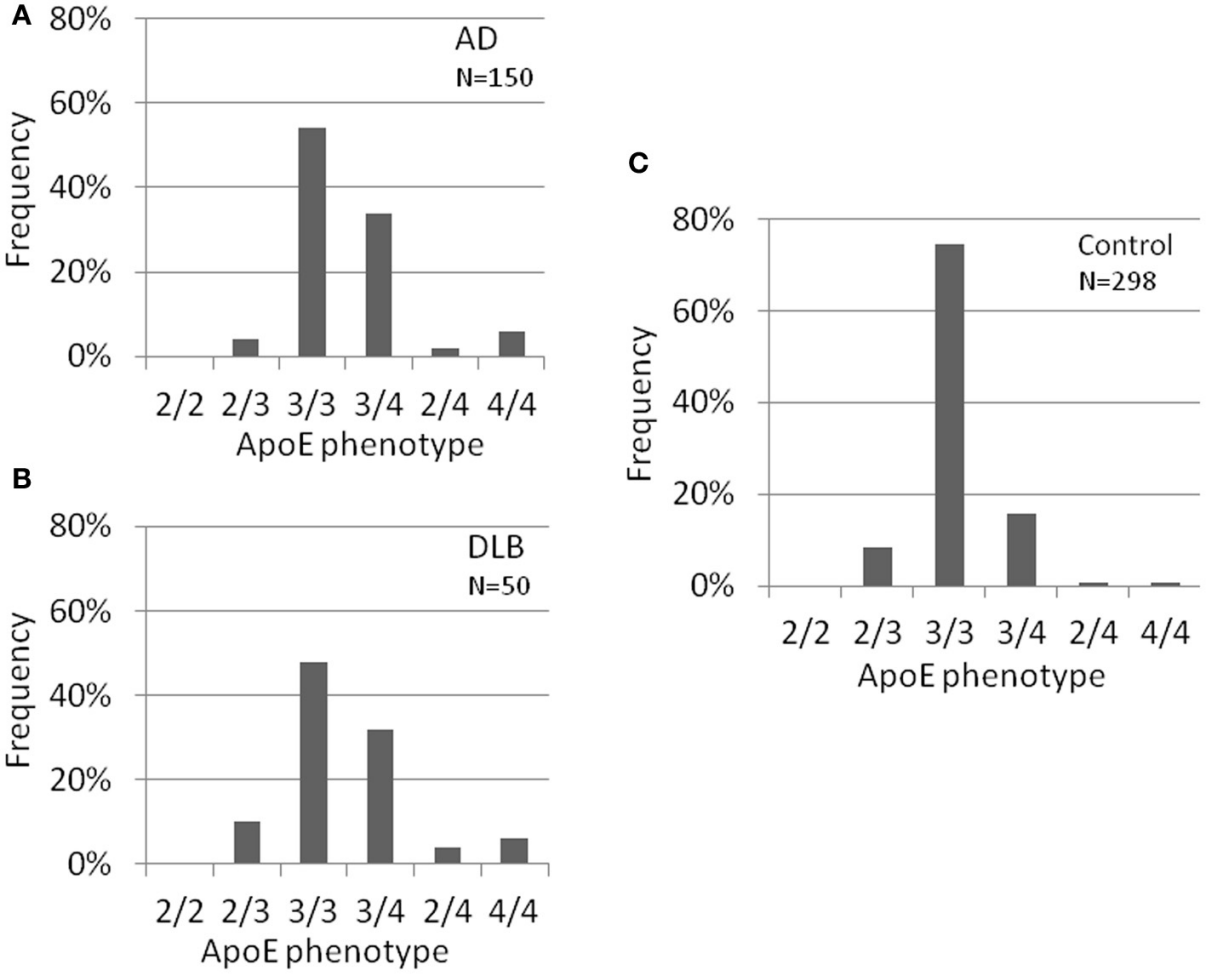
**ApoE carrier statuses of AD (A), DLB (B), and healthy volunteers (control) (C).** ApoE carrier statuses are significantly different between AD and control, and between DLB and control. However, no significant differences were observed between AD and DLB. ApoE typing was done using pcr and restriction enzyme digestion using genomic DNA. AD, Alzheimer's disease; DLB, dementia with Lewy bodies.

Several risk factors for AD have been suggested such as medical history, life style, environment and genes. Of these, ApoE-ε4 has been noticed as one of the genetic factors. We next identified ApoE gene typing by analyzing the restriction enzyme products of the PCR-amplified ApoE gene as shown in Materials and Methods (Table 1, Figure 3). In the control group, 51 out of 279 subjects were ApoE4 carriers (18.3%). Three subjects were homozygous for the ε4 allele (1.1%) and 48 subjects were heterozygous for the e4 allele (17.2%). The total frequency of the ε4 allele was 9.7%. In the AD group, 63 out of 150 subjects were ApoE4 carriers (42.0%). Nine subjects were homozygous for the ε4 allele (6.0%) and 54 subjects were heterozygous for the ε4 allele (36.0%). The total frequency of the ε4 allele was 24.0%. In the DLB group, 21 out of 50 subjects were ApoE4 carriers (42.0%). Three subjects were homozygous for the ε4 allele (6.0%) and 18 subjects were heterozygous for the ε4 allele (36.0%). The total frequency of the ε4 allele was 24.0%. ApoE4 frequencies were compared among AD, DLB, and control groups (Fisher's exact test). ApoE4 carrier status was significantly different between AD and control groups (*p* < 0.0001), and between DLB and control (*p* = 0.0004). Allele frequencies of ApoE ε4 were significantly higher in AD (*p* < 0.0001) and DLB (*p* < 0.0001) than in the control group. However, there were no significant differences in rates of ApoE4 carrier status (*p* = 0.57) and the frequencies of the ε4 allele (*p* = 0.32) between AD and DLB. These results also indicate the similarity of AD and DLB.

**Table 1:** **Distribution of ApoE4 carrier status and the frequency of ApoE ε4 allele in the population of AD, DLB, and control groups**.

	ApoE4 carrier^*^		ApoE ε4 allele^**^	
	**Positive**	**Negative**	**Positive**	**Negative**
C	51 (18.3%)	228 (81.7%)	57 (10.2%)	501 (89.8%)
AD	63 (42.0%)	87 (58.0%)	72 (24.0%)	228 (76%)
DLB	21 (42.0%)	29 (58.0%)	27 (24.0%)	73 (76.0%)

Numbers in parentheses represent the frequencies of ApoE4 carrier or ApoE ε4 allele. C, control group; AD, Alzheimer's disease group; DLB, dementia with Lewy bodies group.

* Significantly different between AD and control groups (p < 0.0001), and between DLB and control (p = 0.0004).

** Significantly higher in AD (p < 0.0001) and DLB (p < 0.0001) than in the control group. No significant differences in rates of ApoE4 carrier status and the frequencies of the ε4 allele between AD and DLB.

## Discussion

### Similarity of AD and DLB

The toxicity of Aβ is thought to cause neural cell death, which is involved in the pathogenesis AD (Mattson, 2004). Decreased degradation or dyscatabolism of Aβ, presumably related to aging, results in both the accumulation of amyloid beta peptide (Aβ_42_) in the brain and the decreased concentration of Aβ_42_ in CSF. Thus, lowered concentration of CSF Aβ_42_ has been noted as a barometer for AD (Andreasen et al., 2001). AD is the most common neurodegenerative dementia and DLB is the second most common. DLB shares clinical and pathological features with AD, which makes it difficult to distinguish in clinical practice. The CSF levels of Aβ_42_ are similar between AD and DLB (Gomez-Tortosa et al., 2003; Mollenhauer et al., 2005a). Amyloid-dependent neurotoxicity is known to perturb Ca^2+^ homeostasis in neuronal cells (LaFerla, 2002). Possibly, Aβ impairs membrane Ca^2+^ pumps and enhances Ca^2+^ influx through voltage-dependent channels and ionotropic glutamate receptors. Focusing on proteins concerning Ca^2+^ signaling, we identified annexin A5 which is augmented in Aβ_42_ dependent manner and showed it as a potential biomarker for AD (Yamaguchi et al., 2010). Moreover, the plasma level of annexin A5 was shown to be elevated in AD (Yamaguchi et al., 2010). In the present study, plasma level of annexin A5 was shown to be elevated not only in AD but also in DLB.

Genetic factors are increasingly recognized as major risk factors for dementia. Evidence from numerous studies has identified the ApoE gene on chromosome 19 as a major risk factor for AD. ApoE, which is a major component of lipoproteins, is comprised of 299 amino acid residues and plays a role in the metabolism and redistribution of cholesterol (Hatters et al., 2006). Three major common isoforms, designated ApoE2, ApoE3, and ApoE4. ApoE colocalizes with extracellular amyloid deposits, resulting in isoform-specific clearance of Aβ. However, ApoE isoforms differently interact with Aβ isoform specific effects on Aβ-clearance. In ApoE4, domain interaction occurs as a result of a putative salt bridge, leading to tight structural formation. This interaction does not occur to the same extent in ApoE2 and ApoE3 (Dong et al., 1994; Dong and Weisgraber, 1996). ApoE ε4 is associated with an increased risk for AD with an earlier age of disease onset (Kim et al., 2009). On the other hand, findings regarding ApoE polymorphisms in DLB have so far been inconclusive. It was reported that ApoE4 carrier frequency was the highest in AD among AD, DLB, and control groups, and it was higher in DLB than in control groups (Carrillo Garcia et al., 2008). Other findings have shown that ApoE4 carrier and allelic frequencies were comparable for those with AD and DLB [(Kobayashi et al., 2011) and Table 1].

Our results for annexin A5 and ApoE4 also revealed similar characteristics for both AD and DLB patients.

### Difference between AD and DLB

It is apparent that DLB differs from AD in the disease progression and cure response experienced by patients. Accordingly, early differentiation between the two forms of dementia is important for effective and safe management (Aarsland et al., 2008; Sinha et al., 2011). CSF levels of tau protein have been shown to be significantly lower in DLB than in AD, which may help to differentiate between the two diseases (Mollenhauer et al., 2005a,b). On the other hand, another study also suggests that the concentration of phosphorylated tau in CSF, which is highly correlated with total tau levels, may provide a higher specificity to differentiate AD and DLB (Vanderstichele et al., 2006). α-Synuclein is the major constituent of Lewy bodies found in neurons in DLB. As a consequence of increased accumulation of α-synuclein intraneuronally in DLB, several studies have attempted its quantification in CSF. α-Synuclein has been shown to induce disruption of cellular inorganic ion homeostasis such as Ca^2+^, leading to cell death (Lowe et al., 2004; Danzer et al., 2007; Ying et al., 2011). Whereas some groups show a decrease in the total concentration of CSF α-synuclein in DLB in comparison to other dementias (Mollenhauer et al., 2008; Kasuga et al., 2010), other groups do not find the significant difference for DLB (Spies et al., 1998; Noguchi-Shinohara et al., 2009). Thus, future study on the discrimination of these diseases is expected.

### About biomarkers

One of the main focuses of public health is prevention of disease. Different stages in the disease process can be targeted for preventative action, including prior to development of the disease, during the asymptomatic stage, and following clinical diagnosis. Therefore, three stages of prevention can be recognized (Wright et al., 2009; Weber et al., 2012).

From the CSF proteins identification, and MRI and PET imaging studies, the alteration of both CSF biomarkers (Aβ_42_ and Tau) takes place prior to the appearance of brain structural change or dementia symptoms (Jack et al., 2010). Our *in vitro* data demonstrated that annexin A5 is elevated following the stimulation by Aβ_42_ (Yamaguchi et al., 2010). Thus, onset of the annexin A5 elevation in dementia occurs at the similar time to the deposition of Aβ_42_. Annexin A5 might be expected to be useful in the secondary and tertiary stages. It is conceivable that the appropriate stage for utilizing each biomarker candidate is dependent upon the properties of the biomarker. Therefore, to determine when each biomarker candidate should be utilized it will be necessary to examine the significance of any biological changes that appear at various stages.

Discrimination between neurodegenerative and non-neurodegenerative dementia is another expectation for biomarkers. Shared clinical symptoms between AD and depression in elderly have been reported (Starkstein et al., 2005), which might lead to confusion in medical intervention. Our preliminary data suggest that plasma annexin A5 levels of the six patients with depression was comparable with controls (data not shown), which might implicate annexin A5 as a biomarker for discriminating between neurodegenerative and non-neurodegenerative diseases.

Biomarkers should be reliable, reproducible, non-invasive, simple to perform, and inexpensive. To achieve this role both protein-based and genetic biomarkers have been particularly investigated. Especially plasma biomarker is beneficial by being less invasive in comparison with CSF biomarker. Gene typing is also less invasive since it is available with leukocytes from a blood sample and genetic biomarkers are of great use. ApoE4 ε4 is widely recognized as a potential biomarker for the risk of AD. As we demonstrated in this paper, ApoE ε4 is also a risk factor for DLB, indicating that ApoE ε4 is unable to discriminate between AD and DLB. No applicable genetic marker for such purpose has been reported. Detailed molecular mechanism of the onset of both AD and DLB may be needed to explore genetic factors.

### Conflict of interest statement

The authors declare that the research was conducted in the absence of any commercial or financial relationships that could be construed as a potential conflict of interest.
